# Fabrication of Fragment Antibody–Enzyme Complex as a Sensing Element for Immunosensing

**DOI:** 10.3390/ijms23031335

**Published:** 2022-01-25

**Authors:** Miho Oda, Ryutaro Asano

**Affiliations:** 1 Department of Biotechnology and Life Science, Graduate School of Engineering, Tokyo University of Agriculture and Technology, Tokyo 184-8588, Japan; s214199s@st.go.tuat.ac.jp; 2 Institute of Global Innovation Research, Tokyo University of Agriculture and Technology, Tokyo 184-8588, Japan

**Keywords:** antibody enzyme complex, Catcher/Tag system, chemical conjugation, direct fusion, enzymatic conjugation, fragment antibody, immunosensing, single-chain Fv, variable domain of heavy chain of heavy-chain antibody

## Abstract

Antibody–enzyme complexes (AECs) are ideal molecular recognition elements for immunosensing applications. One molecule possesses both a binding ability to specific targets and catalytic activity to gain signals, particularly oxidoreductases, which can be integrated into rapid and sensitive electrochemical measurements. The development of AECs using fragment antibodies rather than intact antibodies, such as immunoglobulin G (IgG), has attracted attention for overcoming the ethical and cost issues associated with the production of intact antibodies. Conventionally, chemical conjugation has been used to fabricate AECs; however, controlling stoichiometric conjugation using this method is difficult. To prepare homogeneous AECs, methods based on direct fusion and enzymatic conjugation have been developed, and more convenient methods using Catcher/Tag systems as coupling modules have been reported. In this review, we summarize the methods for fabricating AECs using fragment antibodies developed for sensing applications and discuss the advantages and disadvantages of each method.

## 1. Introduction

Antibodies have been widely used as sensing elements and for therapeutic purposes because of their high specificity and affinity for target capture [1]. Biosensors utilizing antibodies are referred to as immunosensors and are classified into two types: labeled immunoassays and non-labeled immunoassays, depending on the detection method. In the labeled immunoassay, target molecules are detected using an antibody labeled with a substance showing a binding signal. Fluorescent molecules, such as green fluorescent protein [2,3], nanoparticles such as quantum dots and gold nanoparticles [4,5], and enzymes have been widely used in these applications as substances [6,7]. Among them, enzymes are advantageous because they can easily amplify signals and can be applied for electrochemical detection. Therefore, antibody–enzyme complexes (AECs) have been integrated into immunoassays for clinical diagnoses, such as enzyme-linked immunosorbent assays (ELISA) [8,9,10], chemiluminescence immunoassays [11,12], and immunochromatography [13]. During the COVID-19 pandemic, immunosensors using AECs have also contributed to detection and diagnosis. Anti-SARS-CoV-2 antibodies have been tested by ELISA, immunochromatography [14], and optical/chemiluminescence in saliva and serum [15]. In addition, the development of electrochemical immunosensors for rapid and convenient point-of-care testing (POCT) has been reported. For example, the detection of SARS-CoV-2 spike protein in serum by the multichannel immunoassay platform [16], and the detection of SARS-CoV-2 spike and nucleocapsid protein in saliva by sandwich assay using magnetic beads, have been reported [17].

Alkaline phosphatase (ALP) and horseradish peroxidase (HRP) are often used because of their high enzymatic activities; for example, the reported activities of ALP from calf intestine and HRP are 2500 U/mg and 263 U/mg, respectively [18], which are desirable for labeling [19,20]. These enzymes have also been used as models in immobilization studies [21,22]. In addition, oxidoreductases that can transfer electrons, such as glucose oxidase and glucose dehydrogenase (GDH), are used for electrochemical detection [23,24,25].

**Figure 1:**
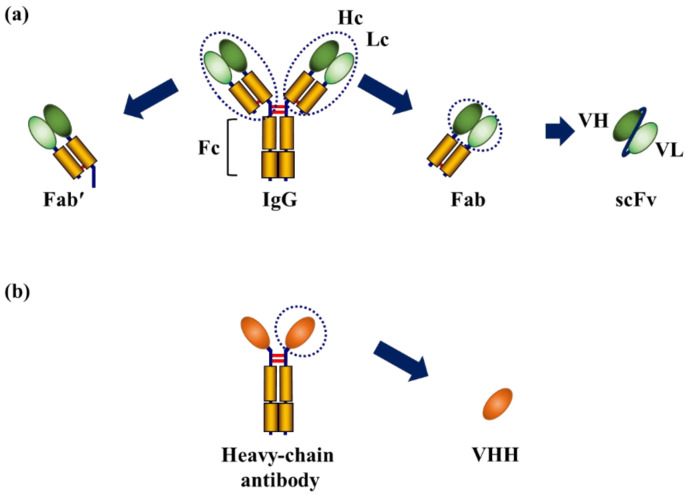
Structure of antibodies and fragment antibodies generally used to fabricate antibody–enzyme complexes (AECs). (**a**) Structure of IgG and fragment antibodies; (**b**) Structure of the variable domain of heavy chain of heavy-chain antibodies (VHH). Fab: fragment antibody-binding; Fab’: Fab with the hinge region; scFv: single-chain fragment variable. Disulfide bonds are presented in red.

To date, most AECs use intact antibodies, particularly immunoglobulin G (IgG) antibodies [26,27,28]. IgG antibodies have a four-chain structure in which two heavy chains (Hc) and two light chains (Lc) are combined with non-covalent and disulfide bonds. IgG antibodies are composed of the fragment antibody-binding (Fab) region and a fragment crystallizable (Fc) region, and the Fab region contains a fragment variable (Fv) region consisting of a heavy chain variable region (VH) and a light chain variable region (VL), which has an antigen-binding ability (Figure 1a). For in vitro sensing, it is not necessary to modify antibodies, such as through humanization, to reduce immunogenicity in humans. Therefore, an efficient and convenient method for the mass production of IgG antibodies is to purify them from the ascitic fluid and blood of hybridoma-inoculated mice.

However, this involves the sacrifice of laboratory animals, which poses ethical issues [29]. Recent recombinant technologies have made it possible to prepare functional fragment antibodies with antigen-recognition sites [30]. In addition to the Fab, Fab with the hinge region (Fab’) as well as a single-chain Fv (scFv), in which the VH and VL of the antibody are linked by an amino acid linker, have been constructed (Figure 1a). Moreover, the variable domain of heavy chain of heavy-chain antibodies (VHH) derived from camelids has also been generally used (Figure 1b). These fragment antibodies can be produced using cost-effective bacterial expression systems without ethical issues. In addition, their small molecular size may contribute to the detection of targets that cannot be accessed by intact antibodies because of steric hindrance.

Several methods have been reported for the fabrication of AECs, such as chemical conjugation, direct fusion, and enzymatic conjugation. In this review, we summarize the sensing elements of AECs from the perspective of the fabrication method, including their advantages and disadvantages, and discuss future prospects.

## 2. Chemical Conjugation

**Figure 2:**
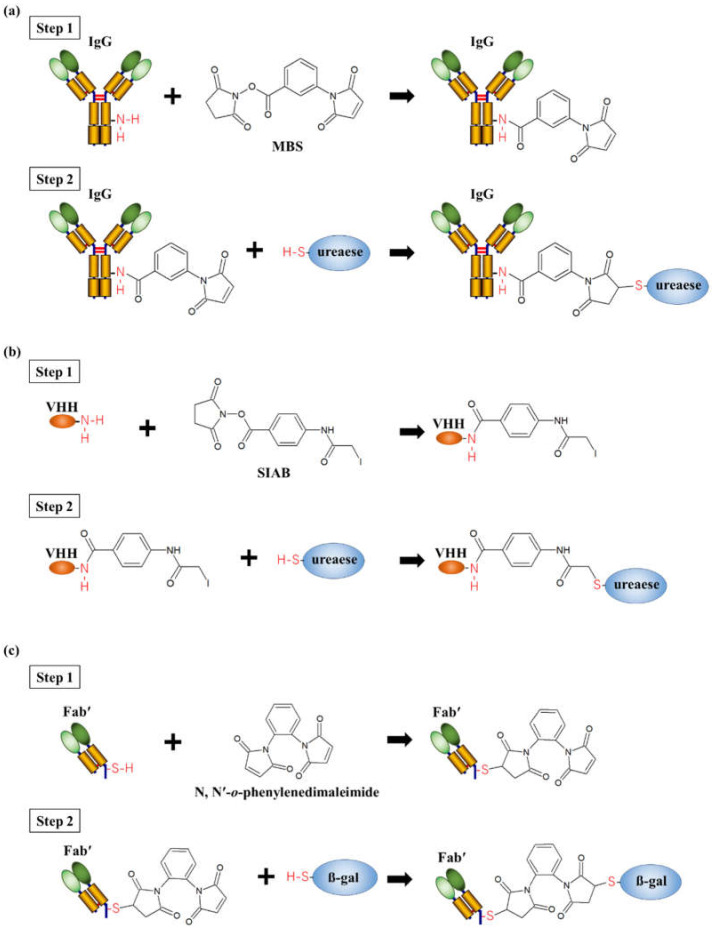
Examples of the fabrication of antibody–enzyme complexes (AECs) by chemical conjugation. (**a**) First step: modification of lysine (Lys) residues in anti-β-bungarotox (β-BuTX) IgG using *m*-maleimidobenzoyl *N*-hydroxysuccinimide (MBS); second step: formation of AECs with MBS-modified IgGs and the thiol group of ureases [31]. (**b**) First step: modification of Lys residues in anti-carcinoembryonic, antigen-related cell adhesion molecule 6 (CEACAM6) VHHs using N-succinimidyl (4-indoacety)aminobenzoate (SIAB); second step: formation of AECs with SIAB-modified VHHs and the thiol group of ureases [32]. (**c**) First step: modification of cysteine residues in anti-human IgG Fab’s using N, N’-*o*-phenylenedimaleimide; second step: formation of AECs with modified Fab’s and the thiol group of β-gals [33].

Chemical conjugation is a conventional approach used to cross-link proteins and is mainly conducted using two methods that involve the targeting of lysine (Lys) or cysteine (Cys) residues on proteins. For sensing, Lys residues on an IgG antibody can be conjugated by an enzyme. Selvanayagam et al. fabricated an AEC consisting of anti-β-bungarotox (β-BuTX) IgGs and urease to detect snake envenomation β-BuTX [31]. Using *m*-maleimidobenzoyl *N*-hydroxysuccinimide (MBS) as a cross-linking agent, a maleimide group was added to the anti-β-BuTX IgG and reacted with the thiol group of Cys residues on the urease for conjugation (Figure 2a). The ion-sensitive field-effect transistor (ISFET)-based electrochemical detection of β-BuTX was successfully performed using the fabricated AEC, which can detect target concentrations as low as 15.6 ng/mL. AECs have also been fabricated using fragment antibodies via chemical conjugation by targeting Lys residues for therapeutic purposes; however, there are no reports describing this approach to sensing. Tian et al. conjugated anti-carcinoembryonic, antigen-related cell adhesion molecule 6 (CEACAM6) VHH with urease using N-succinimidyl (4-indoacetyl)aminobenzoate (SIAB) forming an AEC that acts as a therapeutic agent for tumors expressing CEACAM6 [32]. The AEC binds to CEACAM6 on the surface of tumor cells, and urease converts endogenous urea into ammonia in situ to induce cytotoxicity. The AEC was prepared through the reaction of the amino group of VHH with the succinimide ester group of SIAB, followed by the reaction of the thiol group of the urease with the indoacetyl group (Figure 2b). These methods do not require the addition of tags or modules, which often negatively affect the activity and/or structure of proteins. However, because there are typically multiple Lys residues on each protein, controlling the stoichiometric conjugation and orientation of antibodies is difficult, which may affect the reproducibility and sensitivity during sensing.

A thiol group in the Cys residue is also commonly used for the chemical conjugation of proteins. Kato et al. fabricated anti-human IgG Fab’ and β-D-galactosidase (β-gal) for the detection of human IgG using an N, N’-*o*-phenylenedimaleimide with two maleimide groups as a cross-linking agent [33]. After reacting the thiol group of Fab’ with the maleimide group of one of the cross-linking agents, the other maleimide group was reacted with the thiol group of β-gal to crosslink Fab’ and β-gal (Figure 2c). Using the AEC, human IgG was successfully detected at a low concentration of 0.3 fmoles. In most cases, there are no free Cys residues on the protein surface, including scFv and VHH; thus, stoichiometric conjugation for AEC can be achieved by introducing a Cys residue through the site-directed mutagenesis of enzymes and/or antibodies. In contrast, the additional Cys residue may lead to unfavorable interchain or intrachain disulfide bonds, resulting in low productivity and heterogeneity.

## 3. Direct Fusion

**Figure 3:**
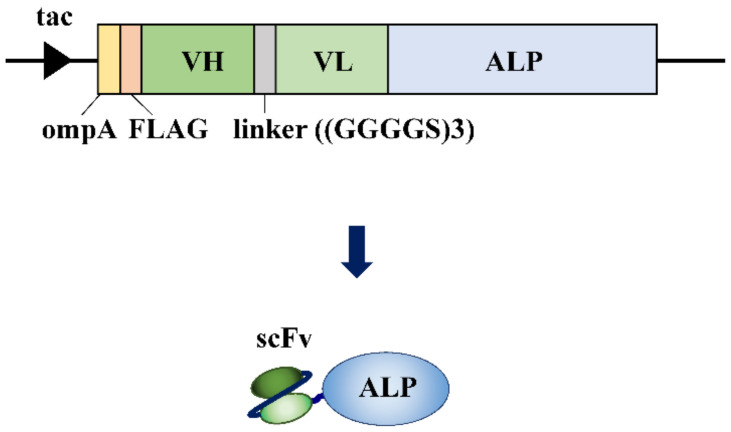
Example of fabrication of antibody–enzyme complex (AEC) by direct fusion. The expression vector for anti-human erb-2 receptor scFv-alkaline phosphatase (ALP) fusion protein contains the isopropylthio-β-galactoside (IPTG) inducible tac promoter, OmpA signal sequence (ompA), and FLAG tag (FLAG) for purification. The AEC was prepared using an *Escherichia coli* expression system [34].

A simple method for fabricating AECs involves the direct genetic fusion of fragment antibodies and enzymes, which has been reported in many studies, including sensing applications. The first report by Wels et al. described an anti-human erbB-2 receptor scFv and ALP fusion for the immunocytochemical detection of human erbB-2 receptors on SKBR3 cells, a human breast cancer cell line that expresses erbB-2 (Figure 3). SKBR3 cells were incubated with fabricated AECs and Fast Red was added as a substrate [34]. The ErbB-2 receptors were successfully stained and detected using the fabricated AECs by immunofluorescence. Carrier et al. developed a fusion product of ALP and anti-human IgG Fab or scFv, which was used to successfully detect human IgG by ELISA [35]. The two AECs enabled the detection of human IgG in the pg/mL range. Koliasnikov et al. reported the fusion of an anti-atrazine Fab and HRP using Pichia pastoris [36]. In this study, HRPs were fused to the C-terminus or N-terminus of Fab to prepare Fab-HRP and HRP-Fab, respectively. In the ELISA, increases in the HRP signal over a wide range of atrazine concentrations from 0.1 to 50 ng/mL were observed using Fab-HRP, whereas HRP-Fab was not effective as a sensing element. These results indicate that the desirable fusion site of the enzyme is the C-terminus of the fragment antibodies. One advantage of direct genetic fusion is that the sensing elements can be prepared in a single production process. In contrast, as in the case of HRP-Fab, because the antibody has an antigen recognition site around the N-terminus, the fusion of enzymes to this region often results in a decrease in or loss of binding ability of the antibody. In addition, aggregates often form during preparation, leading to low production yields because fusion proteins generally have relatively large molecular weights. The strategy for improving detection sensitivity based on the avidity effect through the conjugation of multiple fragment antibodies with one enzyme is difficult to apply to AECs prepared by direct genetic fusion.

## 4. Enzymatic Conjugation

**Figure 4:**
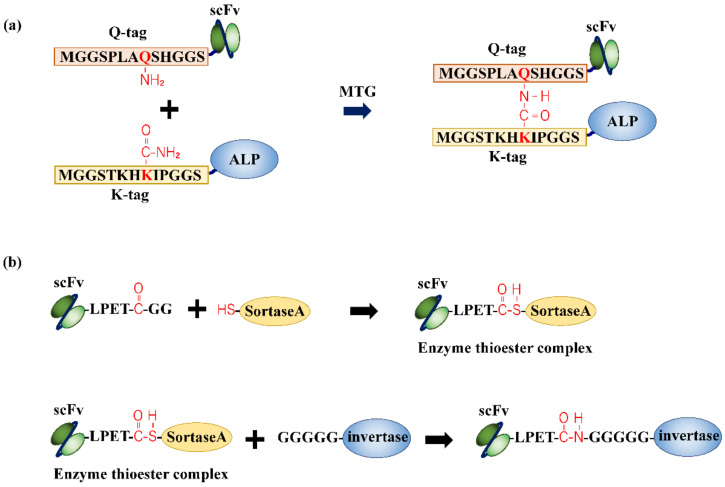
Example of fabrication of antibody–enzyme complexes (AECs) via the catalytic activity of enzymes. (**a**) Q-tag fused anti-hen egg-white lysozyme (HEL) scFv- and K-tag-fused alkaline phosphatase are conjugated via the catalytic activity of microbial transglutaminase (MTG). An amide bond is formed between the glutamine residue of the Q-tag and second lysine residue of the K-tag [37]. (**b**) Sortase A (SrtA) recognizes the LPXTG sequence fused to the C-terminus of anti-ubiquitin scFv, cleaves the bond between the threonine and glycine residues, and forms a thioester intermediate via the cysteine residue of SrtA. The resulting molecule nucleophilically attacks the oligoglycine sequence fused to the N-terminal of invertase to form a peptide bond [39].

Enzymatic conjugation reactions for AEC construction have also been reported. Takazawa et al. fabricated a complex of anti-hen egg-white lysozyme (HEL) scFv and ALP, using microbial transglutaminase (MTG) isolated from *Streptomyces mobaraensis*, to detect HEL, a food allergen [37]. MTG catalyzes the acyl transfer reaction between the γ-carboxamide group of the glutamine (Gln) residue and ε-amino group of the Lys residue to form an ε-(γ-glutamyl) lysine bond [38]. MTG recognizes Gln and Lys residues around the F-helix of Myoglobin. The peptide tags, Q-tag (MGGSPLAQSHGGS) and K-tag (MGGSTKHKIPGGS), were designed based on the F-helix. The Q- and K-tags were genetically fused to the N-terminus of scFv and ALP, respectively. By reacting the Q-tag fusion scFv and K-tag fusion ALP with MTG for 3 h at 4 °C, an ε-(γ-glutamyl) lysine bond between the Gln residue of the Q-tag and Lys residue of the K-tag was formed via the catalytic reaction of MTG (Figure 4a). As a result, HEL concentrations greater than 1 ng/mL were detected using the fabricated AEC. Unlike chemical conjugation, MTG recognizes only Gln and Lys residues in a specific amino acid sequence and does not promote cross-linking between other Gln and Lys residues in proteins. However, MTG also recognizes an N-terminal primary amine, which can lead to the generation of by-products, such as oligomers. Ismail et al. utilized sortase A (SrtA) to fabricate a complex of an anti-ubiquitin scFv and invertase, an enzyme that hydrolyzes sucrose, to detect ubiquitin as a model protein [39]. SrtA is a peptide transferase derived from *Staphylococcus aureus* that catalyzes the binding of the microbial surface component, which is a virulent factor that recognizes adhesive matrix molecules to lipid II of the Gram-positive cell wall. SrtA recognizes the LPXTG sequence (X is an arbitrary amino acid), cleaves the bond between threonine (Thr) and glycine residues, and forms a thioester intermediate via the Cys residue of SrtA. Next, it nucleophilically attacks the oligoglycine sequence to form a peptide bond [40]. Anti-ubiquitin scFv with a C-terminal LPETGG sequence was successfully conjugated to invertase with an N-terminal GGGGG sequence in a catalytic reaction involving SrtA (Figure 4b). Ubiquitin levels of 0.03 pmol to 0.003 fmol were detected by ELISA using the AEC and a commercially available personal glucose meter that could measure the glucose produced from the hydrolysis of sucrose by invertase. Although the reaction time was relatively short (~3 h), this method had some limitations. The recognition sequences, LPETGG and oligoglycine, must be at the C-terminus of one target protein and the N-terminus of the other target protein, respectively. In addition, the conjugation rate is relatively low because of the reverse reaction, which reaches equilibrium after 50% conversion [41].

## 5. Catcher/Tag System

**Figure 5:**
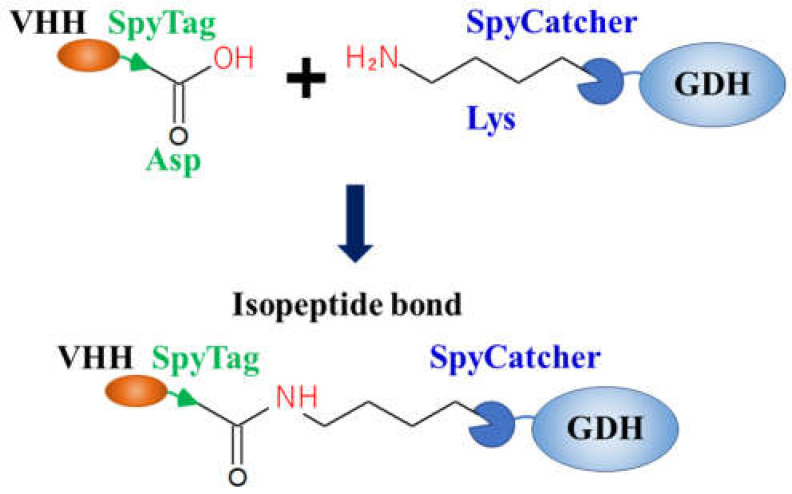
Example of fabrication of AEC via the SpyCatcher/SpyTag system. The isopeptide bond formed between aspartate residue of the SpyTag fused to the anti-epidermal growth factor receptor VHH, and the lysine residue of the SpyCatcher fused to glucose dehydrogenase under mild conditions [42].

To overcome the limitations of the conventional method, we developed a new method for fabricating AECs using the Catcher/Tag systems, which spontaneously form irreversible covalent bonds by mixing protein domains (Catchers) with each counter peptide tag (Tags). We first fabricated a complex of anti-epidermal growth factor receptor (EGFR) VHH and GDH using the SpyCatcher/SpyTag system (Figure 5) [42]. The SpyCatcher/SpyTag is generated from the CnaB2 domain of the fibronectin adhesion protein FbaB derived from *Streptococcus pyogenes*, forming an isopeptide bond between Lys31 of SpyCatcher and aspartic acid (Asp)117 of SpyTag spontaneously and covalently under mild conditions [43]. Three types of complexes were prepared using the SpyCatcher N-terminus, C-terminus, or NC-terminus fusion GDH and SpyTag C-terminus fusion anti-EGFR VHH, and the binding abilities of all AECs were confirmed using surface plasmon resonance analysis. However, in ELISA, EGFR was detected only when the bivalent AEC, that is, the combination of NC terminus fusion GDH and VHH, was used as a sensing element. As described above, the binding site of antibodies is near the N-terminus; in this case, the genetic fusion of GDH to the N-terminus of anti-EGFR VHH resulted in a substantial reduction in affinity. In contrast, our method using the Catcher/Tag systems can keep the N-terminus of the antibody free. Thus, both VHHs in the bivalent AEC functioned cooperatively to enhance affinity and as an effective sensing element. However, the affinities were still insufficient to meet the clinically required detection range of EGFR, as bivalent AECs are inefficient at low concentrations for monomeric targets such as EGFR. To improve affinity, we developed bispecific AECs with two antibody fragments showing different specificities by combining the SpyCatcher/SpyTag and SnoopCatcher/SnoopTag systems, which did not express cross-reactivity. The detection sensitivity for EGFR was improved and the detection range using the bispecific AECs was 0.079–5.0 nM, which met the clinically required EGFR range [44].

In addition, by combining this approach with magnetic separation, we achieved rapid and wash-free homogeneous electrochemical detection of EGFR in the serum by chronoamperometry [44] and confirmed the universality of our method by detecting another target: human hemoglobin [45].

## 6. Conclusions

In this review, we summarize AECs designed as sensing elements based on the fabrication method (Table 1). The characteristics of each fabrication method for AEC are summarized in Table 2. Although chemical conjugation does not require the addition of tags or modules, it is difficult to control the stoichiometric conjugation of antibodies and enzymes, because all Lys and Cys residues in the protein may participate in crosslinking. Additionally, there are concerns regarding the effects of chemical cross-linking agents on the structure and activity of proteins. In the direct genetic fusion method, because antibodies have an antigen recognition site around the N-terminus, the fusion of enzymes to the N-terminus of antibodies often results in a decrease in or loss of binding abilities, making it difficult to design AECs with multiple antibodies. In enzymatic conjugation, the method using MTG cannot produce stoichiometric AECs, which is similar to chemical conjugation. The crosslinking site of SrtA is restricted because the recognition sequences must be at the C-terminus of one target protein and the N-terminus of the other target protein. The reaction efficiency was relatively low owing to the reversibility of the reaction. In contrast, the Catcher/Tag systems can stoichiometrically conjugate antibodies and enzymes with an irreversible covalent bond. Although the Catcher/Tag domain derived from a microorganism remains between an antibody and an enzyme after conjugation, immunogenicity is irrelevant when AECs are used for in vitro sensing. However, immunogenicity may be problematic when developing a continuous monitoring system using an implantable device and AECs prepared using the Catcher/Tag systems. Utilizing the Catcher/Tag systems, which can easily produce various AECs by simply mixing Catcher-fused enzymes and Tag-fused antibodies, may be suitable for screening combinations of enzymes and antibodies that can detect each target. As mentioned above, the preparation of AECs with multiple antibodies is difficult using the direct genetic fusion method. However, this method enables the preparation of AECs in a single production process, unlike the method using the Catcher/Tag systems. For the ALP C-terminus fusion scFv, a higher affinity was observed compared with scFv alone, and a dimeric structure of ALP was useful for preparing AECs with two scFvs [46]. Thus, an ideal strategy for developing functional and practical AECs may be using a direct genetic fusion method and enzymes with multimeric structures after screening, in order to obtain good combinations of enzymes and antibodies using the Catcher/Tag systems.

## Figures and Tables

**Table 1:** Comparison of immunosensors using AEC.

Fabrication Method	Antibody Format	Enzyme	Detection Target	Detection Method	Detection Limit	Reference
Chemical conjugation	IgG	Urease	β-BuTX	ELISA (ISFET)	15.6 ng/mL	[31]
	Fab’	β-gal	Human IgG	ELISA	0.3 fmoles (45 pg *)	[33]
Direct fusion	scFv	ALP	Human erbB-2 receptors	Immunostaining (Fluorescence)	-	[34]
	Fab/scFv	ALP	Human IgG	ELISA	No data	[35]
	Fab	HRP	Atrazine	ELISA	0.1 ng/mL	[36]
Enzymatic conjugation	scFv	ALP	HEL	ELISA	1 ng/mL	[37]
	scFv	Invertase	Ubiquitin	ELISA	0.003 fmol (26 fg *)	[39]
Catcher/Tag system	VHH	GDH	EGFR	ELISA	3.75 nM (260 ng/mL *)	[42]
	scFv	GDH	EGFR	ELISA (Fluorescence) ELISA (Amperometry) ELISA (Amperometry, Homogeneous)	0.079 nM (5.5 ng/mL *) 0.44 nM (31 ng/mL *) 0.2 nM (14 ng/mL *)	[44]
	scFv	GDH	Hemoglobin	ELISA (Amperometry, Homogeneous)	0.54 nM (35 ng/mL *)	[45]

* Values were converted using estimated molecular weights.

**Table 2:** Characteristics of each fabrication method for AEC.

		Conjugation Efficiency	Reaction Condition	Stoichiometric Conjugation	Note
Chemical conjugation		High	0.5~3 h, 25 °C	No	No/Less protein engineering
Direct fusion		-	-	Yes	Single production
Enzymatic conjugation	MTG	Middle	3 h, 4 °C	No	Less impact on protein function
	SrtA	Low	3 h, 25 °C	Yes	
Catcher/Tag system		High	1~40 h, 4 °C	Yes	High versatility
